# Diagnosis of Pneumoperitoneum with Bedside Ultrasound

**DOI:** 10.5811/westjem.2014.12.24945

**Published:** 2015-02-25

**Authors:** Alice Chao, Laleh Gharahbaghian, Phillips Perera

**Affiliations:** Stanford University, Department of Emergency Medicine, Stanford, California

An 86-year-old female was brought in by ambulance for severe abdominal and back pain. She was hypotensive en route and appeared to be in distress upon arrival to the emergency department. Her abdomen was tense and distended with diffuse tenderness to palpation present. A bedside abdominal ultrasound (US) was done immediately, which raised concern for free air. A portable upright chest x-ray was obtained, which confirmed the diagnosis of pneumoperitoneum (Video).

Pneumoperitoneum due to perforated viscus is an emergent diagnosis that requires immediate surgical consultation and intervention. US is a useful tool that can be done at the bedside to rapidly make the diagnosis. Both low- and high-frequency transducers may be used to detect intraperitoneal free air. With the patient in a supine position, the perihepatic space should be evaluated. The patient may also be turned to the left lateral decubitus position to facilitate the rise of free air to the RUQ.^1^ Findings may also be seen from the anterior abdominal wall when the patient is supine. Pneumoperitoneum can be detected on US by the enhanced peritoneal stripe sign (EPSS) in conjunction with reverberation artifacts, which have the appearance of repeating linear lines extending at equidistant distances posteriorly from the peritoneal lining.^2^ Comet tail artifact may also be appreciated from the peritoneal stripe. The “scissors maneuver” can increase sensitivity of US in detecting intraperitoneal free air.^3^ Indirect signs may also be seen on US, including thickened bowel loops or air bubbles in peritoneal free fluid.^4^

US has been shown to have a sensitivity of 85% and a specificity of 100% for pneumoperitoneum. It has been shown by some to have a higher sensitivity for this diagnosis as compared to plain radiography.^5^ Although computed tomography imaging is still the gold standard for pneumoperitoneum, US is a helpful initial diagnostic tool that can be done rapidly at the bedside.

## Figures and Tables

**Video:** Pneumoperitoneum.
